# A two-stage genome-wide association study identifies novel germline genetic variations in CACNA2D3 associated with radiotherapy response in nasopharyngeal carcinoma

**DOI:** 10.1186/s12967-022-03819-4

**Published:** 2023-01-09

**Authors:** Lu-Lu Yu, Bi-Wen Hu, Han-Xue Huang, Bing Yu, Qi Xiao, Qiao-Li Lv, Chen-Hui Luo, Cheng-Xian Guo, Jin-Gao Li, Xiao-Xue Xie, Ji-Ye Yin

**Affiliations:** 1 Department of Clinical Pharmacology, Xiangya Hospital, Central South University, Changsha, 410078 People’s Republic of China; 2 Institute of Clinical Pharmacology, Hunan Key Laboratory of Pharmacogenetics, Central South University, 410078 Changsha, People’s Republic of China; 3 Engineering Research Center of Applied Technology of Pharmacogenomics, Ministry of Education, 110 Xiangya Road, Changsha, 410078 People’s Republic of China; 4 National Clinical Research Center for Geriatric Disorders, 87 Xiangya Road, Changsha, 410008 Hunan People’s Republic of China; 5 Center of Clinical Pharmacology, The Third Xiangya Hospital, Central South University, Changsha, 410013 Hunan People’s Republic of China; 6 Department of Radiation Oncology, Jiangxi Cancer Hospital of Nanchang University, Nanchang, 330029 People’s Republic of China; 7 National Health Commission (NHC) Key Laboratory of Personalized Diagnosis and Treatment of Nasopharyngeal Carcinoma, Jiangxi Cancer Hospital of Nanchang University, Nanchang, 330029 People’s Republic of China; 8 Scientific Research Office, Hunan Cancer Hospital, The Affiliated Cancer Hospital of Xiangya School of Medicine, Central South University, Changsha, China; 9 Department of Radiotherapy, Hunan Provincial Tumor Hospital and Affiliated Tumor Hospital of Xiangya Medical School, Central South University, Changsha, 410013 People’s Republic of China; 10 Department of Radiation Oncology, Hunan Cancer Hospital, Affiliated Hospital of Xiangya Medical School, Central South University, Changsha, 410013 People’s Republic of China

**Keywords:** Genome-wide association study, Nasopharyngeal carcinoma, Radiotherapy, Efficacy, CACNA2D3

## Abstract

**Background:**

Radiotherapy (RT) is the standard treatment for nasopharyngeal carcinoma (NPC). However, due to individual differences in radiosensitivity, biomarkers are needed to tailored radiotherapy to cancer patients. However, comprehensive genome-wide radiogenomic studies on them are still lacking. The aim of this study was to identify genetic variants associated with radiotherapy response in patients with NPC.

**Methods:**

This was a large‑scale genome-wide association analysis (GWAS) including a total of 981 patients. 319 individuals in the discovery stage were genotyped for 688,783 SNPs using whole genome-wide screening microarray. Significant loci were further genotyped using MassARRAY system and TaqMan SNP assays in the validation stages of 847 patients. This study used logistic regression analysis and multiple bioinformatics tools such as PLINK, LocusZoom, LDBlockShow, GTEx, Pancan-meQTL and FUMA to examine genetic variants associated with radiotherapy efficacy in NPC.

**Results:**

After genome-wide level analysis, 19 SNPs entered the validation stage (P < 1 × 10^− 6^), and rs11130424 ultimately showed statistical significance among these SNPs. The efficacy was better in minor allele carriers of rs11130424 than in major allele carriers. Further stratified analysis showed that the association existed in patients in the EBV-positive, smoking, and late-stage (III and IV) subgroups and in patients who underwent both concurrent chemoradiotherapy and induction/adjuvant chemotherapy.

**Conclusion:**

Our study showed that rs11130424 in the CACNA2D3 gene was associated with sensitivity to radiotherapy in NPC patients.

*Trial registration number:* Effect of genetic polymorphism on nasopharyngeal carcinoma chemoradiotherapy reaction, ChiCTR-OPC-14005257, Registered 18 September 2014, http://www.chictr.org.cn/showproj.aspx?proj=9546.

**Supplementary Information:**

The online version contains supplementary material available at 10.1186/s12967-022-03819-4.

## Introduction

Nasopharyngeal carcinoma is a malignant tumor arising from the endothelium of the nasopharynx [1] Its incidence is highly geographically specific, with a high prevalence in countries of China, India and Vietnam. China accounted for approximately half of all new cases of NPC worldwide in 2018 [2].

The main treatment for NPC is radiotherapy [3] The current radiation treatment strategy for NPC is to use simple radiotherapy for early-stage NPC and concurrent chemoradiotherapy for intermediate and advanced stages [4] Although radiotherapy is the major treatment for NPC, there is some individual difference in the outcome of patients receiving radiotherapy, and the exact mechanism has not yet been fully understood. There is currently a problem of over- or under-treatment in clinical care as there is no good way to accurately identify patients who may benefit from radiotherapy prior to treatment. In the treatment of NPC, people seek to improve the efficacy of radiotherapy and increase patient survival.

With regard to the development of biomarkers related to the efficacy of radiotherapy, the concept of radiogenomics has been proposed [5] Radiogenomics is based on the theory that the efficacy of radiotherapy is not only related to the treatment scheme, radiation dose and irradiation location, but also to the genetic characteristics of the patients. Candidate gene association and genome-wide association analysis are the main approaches in radiogenomics studies. Most current studies about biomarkers of tumor radiotherapy efficacy are based on candidate gene association studies. For example, X-ray repair cross complementing 1 (XRCC1) 399Gln was found to be significantly associated with radiotherapy resistance in patients with oesophageal carcinoma [6]; Epidermal growth factor receptor (EGFR) R497K can be used as a predictor of short-term efficacy and late recurrence or metastasis of radiotherapy in cervical cancer [7] In addition, rs3135001 is a useful biomarker for predicting for the sensitivity of head and neck squamous cell carcinoma to radiotherapy [8] However, there are few GWAS on NPC radiotherapy sensitivity, but it is equally important to explore the genetic variants associated with NPC radiotherapy sensitivity.

In this study, we conducted a two-stage GWAS study in 1166 NPC patients to comprehensively examine the entire genome at a higher resolution than candidate gene association studies. We selected multiple NPC susceptibility-associated loci and validated these findings in two additional cohort of samples. Our findings clearly indicate that the CACNA2D3 gene plays a role in the mechanism of radiotherapy sensitivity in NPC.

## Method

### Study population and data collection

All patients in this study were recruited from Jiangxi Provincial Cancer Hospital and Hunan Provincial Cancer Hospital from 2014 to 2022 respectively, who were diagnosed with NPC by pathological histological examination, while having no distant metastases and no other concurrent tumors. They are mainly treated by Intensity Modulated Radiation Therapy (IMRT), which allows for more precise application of different doses of radiation to different target areas. The radiation dose depends on the total tumor volume (GTV), the clinical target volume (CTV) and the planned target volume (PTV). A single dose of radiation therapy is usually about 2 Gy, once a day, 5 days a week. Primary lesions and positive cervical lymph nodes received 66–77 Gy and 54–60 Gy, respectively, and patients were treated in approximately 30–33 sessions. The patients have not received surgery, targeted therapy, immunotherapy or other anti-tumor treatment prior to radiotherapy. Cisplatin was used as the base regimen for adjuvant chemotherapy, neoadjuvant chemotherapy or concurrent radiotherapy. Exclusion criteria are: (1) breastfeeding pregnant patients; (2) having serious complications.

Clinicians used the WHO Response Evaluation Criteria in Solid Tumors (RECIST) to assess the efficacy of each patient after radiotherapy based on the patient’s MRI medical records. In this study, post-radiotherapy efficacy was assessed for the primary lesion and positive lymph nodes. The results of radiotherapy efficacy assessment were classified into four categories: complete remission (CR), partial remission (PR), stable disease (SD) and progressive disease (PD). For association analysis, all patients were further divided into two groups: the group with good outcome as CR, and the group with poor outcome as non-CR (including PR, SD and PD).

### Ethics statement

The study protocol was approved by the Ethics Committee of Jiangxi Cancer Hospital and Hunan Cancer Hospital. Clinical information of all patients from both hospitals were collected with the same protocol and were not any overlap of patients. The ethical aspects of this study are in line with the Declaration of Helsinki. All subjects were provided with written informed consents. We applied this study for clinical admission in the Chinese Clinical Trial Register (registration number: ChiCTR-OPC-14005257).

### Genotype detection, quality control and imputation

For discovery stage samples, genotyping was performed using the Illumina Infinium Global Screening Array-24 v1.0 (GSA) microarray, and the data generated by the Illumina microarray platform was visualized and analyzed according to GenomeStudio software. For quality control, the raw data were first selected for sample and SNP selection using PLINK (version 1.9) software. SNP quality control (QC) conditions: (1) SNP call rate ≥ 95%; (2) HWE ≥ 1 × 10^− 4^; (3) MAF ≥ 0.01; (4) localization on autosomes. Sample QC conditions: (1) Sample call rate ≥ 95%; (2) Correcting for Cryptic relatedness. Cryptic relatedness refers to unknown more recent relatedness including family relationships such as grandparent-grandchild and full sibling pairs; (3) The heterozygosity rate was less than three standard deviations from the mean of all participants; (4) Genotype-estimated sex was discordant with biological sex. Using 1000 Genomes stage 3 v4 as the reference panel, IMPUTE2 was used for genotype imputation. SNPs with INFO below 0.4 and MAF<0.05 were excluded. Finally, 4,112,760 unique SNPs were included in the GWAS analysis. For samples in the validation stage 1, genotyping was performed using the Sequenom MassARRAY system, and genotyping data were obtained using TYPER 4.0 software. Exclusion criteria at this stage included (1) Sample call rate ≥ 95%; (2) SNP detection rate ≥ 95%; (3) MAF ≥ 0.01; (4) HWE ≥ 0.05; (5) Sample relatedness check; (6) The heterozygosity rate was less than three standard deviations from the mean of all participants; and (7) Genotype-estimated sex was discordant with biological sex. For samples in the validation stage 2, genotyping was conducted using the TaqMan SNP assays (Applied Biosystems, Foster City, CA) with a Roche LightCycler 480 Real-Time PCR System (Roche, Basle, Switzerland) for rs11130424. The amplification reaction was carried out in 10 µL of the PCR mixture containing 5 µL of LightCycler® 480 Probes Master (Roche, Switzerland), 0.5 µM of forward primer rs11130424-F: 5′- ATCATGGACGGTCCTTGCT-3′ and 0.5 µM of the reverse primer rs11130424-R: 5′- GGACATGTCAATTTAAAGTTGCTAT − 3′, 0.4 µM of each allele-specific probes (rs11130424_G: 5′-FAM- AAACTCTGTTACTCATAGAA -MGB-3′ and rs11130424_T:5′-VIC- AACTCTGTTATTCATAGAAT -MGB-3′) and 10 ng of DNA. PCR was carried out using a LightCycler® 96 System (Roche, Switzerland) under optimized conditions (preliminary denaturation – 94 ℃–3 min; 40 cycles: 94 ℃–5 s, 60 ℃–30 s). Fluorescence measurements were carried out at the annealing step of PCR in FAM and VIC channels. The results of genotyping were analyzed using the LightCycler® 96 thermal cycler software.

### Genome-wide association analysis

Principal component analysis (PCA) was conducted on all patients in the discovery stage using the SNPRelate procedure. The distribution of the samples was plotted using the R package “ggplot2”. Samples that deviated significantly from the overall were removed and then reconducted for PCA. Clinical factors that have a significantly effect on the phenotype were considered as covariates. T-tests were used for continuous variables and logistic tests for categorical variables, and clinical factors that differed markedly in subgroups were selected as covariates for correction. The significantly associated clinical factors in multivariate regression were adjusted in genome-wide association analyses. For two phenotypes of primary lesion and positive lymph node efficacy, genome-wide association analyses were performed under additive genetic effects assumption, using a logistic regression model adjusting sex, smoking and drinking as covariates. Quantitative-Quantitative plots and Manhattan plots were plotted via the R package “qqman”. A genomic inflation factor λ was calculated for population stratification using PLINK (version 1.9). The deviation of the observed versus the expected distribution of the P values was represented by the inflation factor λ. The closer λ was to 1, the less stratified the population was considered to be.

### Bioinformatics tools

Regional plot was generated using LocusZoom (http://locuszoom.sph.umich.edu/). The LD and haplotype block estimations analyses were performed using PLINK and LDBlockShow (1.35 version) [9]. Expression quantitative trait loci (eQTL) and DNA methylation quantitative trait loci (meQTL) were analyzed using Genotype-Tissue Expression (GTEx) project (https://gtexportal.org/) and Pancan-meQTL database (http://bioinfo.life.hust.edu.cn/Pancan-meQTL/) respectively [10]. Functional annotation of GWAS results and chromatin interaction visualization were generated using the online tool functional mapping and annotation (FUMA) v1.3.4c (https://fuma.ctglab.nl/) [11, 12]. Transcription regulation was predicted based on the data of ENCODE database (https://www.encodeproject.org/) [13]. 1000Genomes project (https://www.ncbi.nlm.nih.gov/variation/tools/1000genomes/) and the gnomAD database (http://gnomad-sg.org/) were used to query the racial frequencies of loci in each ethnicity, respectively.

### Statistical analysis

Pearson’s chi-square test or Fisher’s exact test was used to assess the relationship between clinical characteristics and radiotherapy response. The influences of genetic variants on the radiotherapy sensitivity were examined using multivariable logistic regression by calculating odds ratios (ORs) and their 95% confidence intervals (CIs). In this study, all P values were two-sided, and P < 0.05 was considered statistically significant. All these analyses were performed by PLINK (1.9 version), SPSS (20.0 version), and R v0.4.0.3 (www.r-project.org).

## Result

### Clinical characteristics

**Fig. 1:**
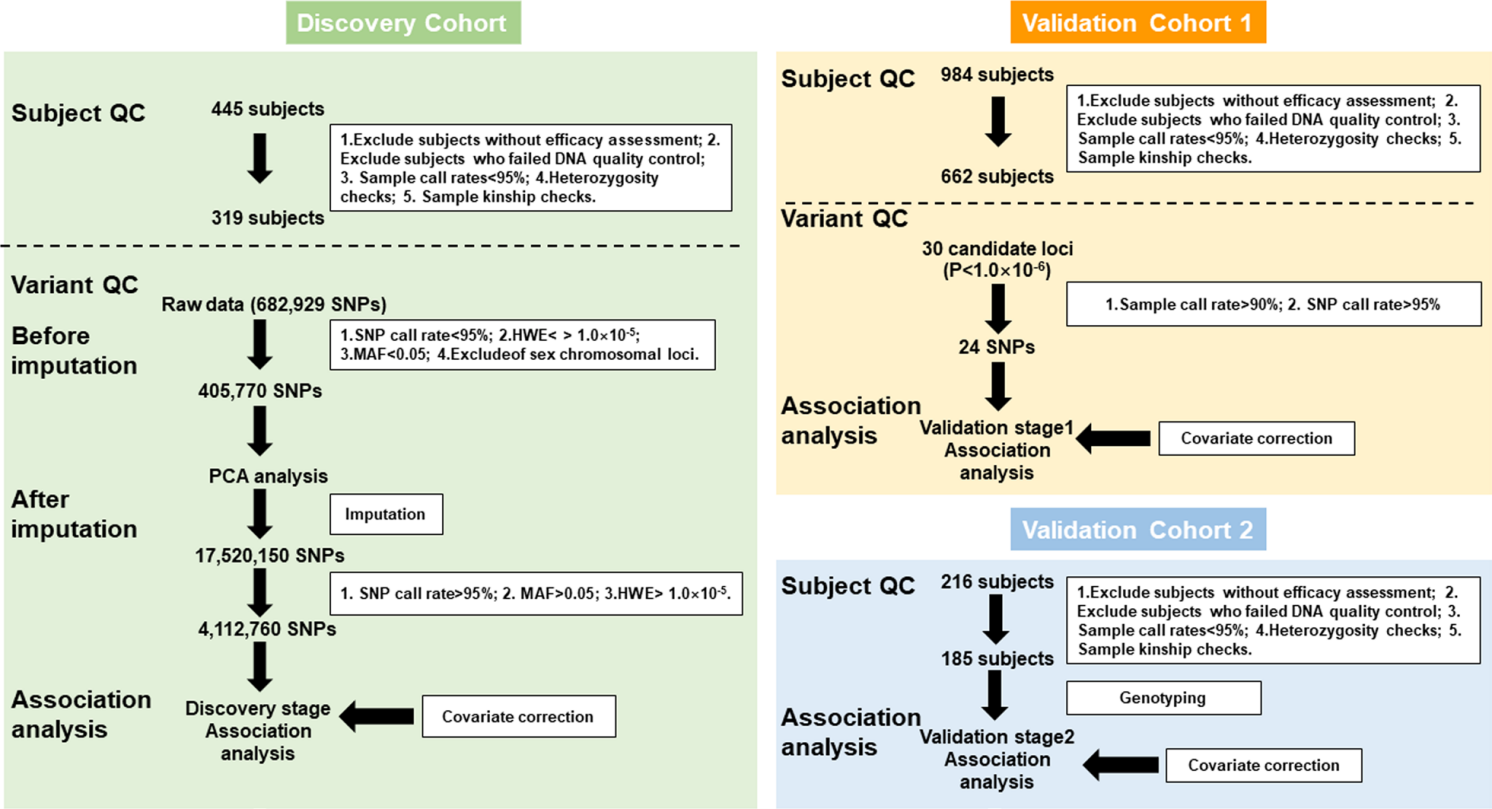
Flow diagram of genome-wide association analysis study design

As shown in Fig. 1, this was a two-stage GWAS study. The study population initially consisted of 1645 patients with NPC and 479 were excluded due to failure of sample quality control. Ultimately, 1166 patients were included in the two-stage analysis. Of these, 319 patients from the Jiangxi Provincial Cancer Hospital were recruited into the discovery phase cohort. 662 patients from Hunan Cancer Hospital and 185 patients from Jiangxi Cancer Hospital were used to validate the study results. Detailed clinical characteristics are shown in Table 1. Here we analyzed 2 indicators of primary lesions and positive lymph node efficacy after radiotherapy. The characteristics of NPC patients involved in these association analyses are summarized in Additional file 1: Tables S1 and S2. For each phenotype, the association of clinical characteristics of age, gender, BMI, smoking status, EBV infection, clinical stage, and treatment scheme between different subgroups was analyzed. All significant clinical factors were considered as covariates and adjusted in subsequent GWAS analysis.

**Table 1:** Characteristics of all patients

Characteristics	Discovery stage	Validation stage 1	Validation stage 2
Number of patients	319	662	185
Age (Mean ± SD)	50.75 ± 11.96	47.45 ± 10.49	51.26 ± 12.13
Gender			
Male	215	488	131
Female	104	173	54
BMI (Mean ± SD)	22.61 ± 3.40	23.18 ± 3.26	23.53 ± 3.27
Smoking status			
Smoker	151	320	74
Non–smoker	168	334	17
NR	0	7	94
Drinking status			
Drinker	72	227	35
Non–drinker	247	427	21
NR	0	7	129
Clinical stage			
I	7	41	1
II	26	55	12
III	120	302	93
IV	165	258	79
NR	1	5	0
EBV			
Positive	216	436	90
Negative	103	219	95
NR	0	6	0
Treatment			
RT alone	60	12	20
RT + IC/AC	4	39	19
CCRT	100	23	23
CCRT + IC/AC	154	551	123
RT + other treatment*	1	36	0

*SD* Standard deviation, *BMI* Body mass index, *EBV* Epstein-Barr virus, *NR* Not reported, *RT* Radiotherapy, *IC* Induction chemotherapy, *AC* adjuvant chemotherapy, *CCRT* Concurrent chemoradiotherapy

*Other treatment included surgery, targeted therapy, immunotherapy and herbs treatment

### Data pre-processing

In the discovery stage, peripheral blood DNA samples from 319 patients were subjected to genome-wide microarray analysis and 688,783 autosomal SNPs were included in the association analysis; in the validation stage, peripheral blood DNA samples from 847 patients were subjected to mass array microarray and TaqMan SNP assays analysis of candidate loci. For each sensitivity phenotype, associations between clinical characteristics such as age, sex, Body Mass Index (BMI), smoking status, EBV infection, clinical stage and treatment regimen were analyzed between different subgroups. All clinical factors that would have an impact were considered as covariates and were adjusted for in subsequent association analyses.

### GWAS analysis

**Fig. 2:**
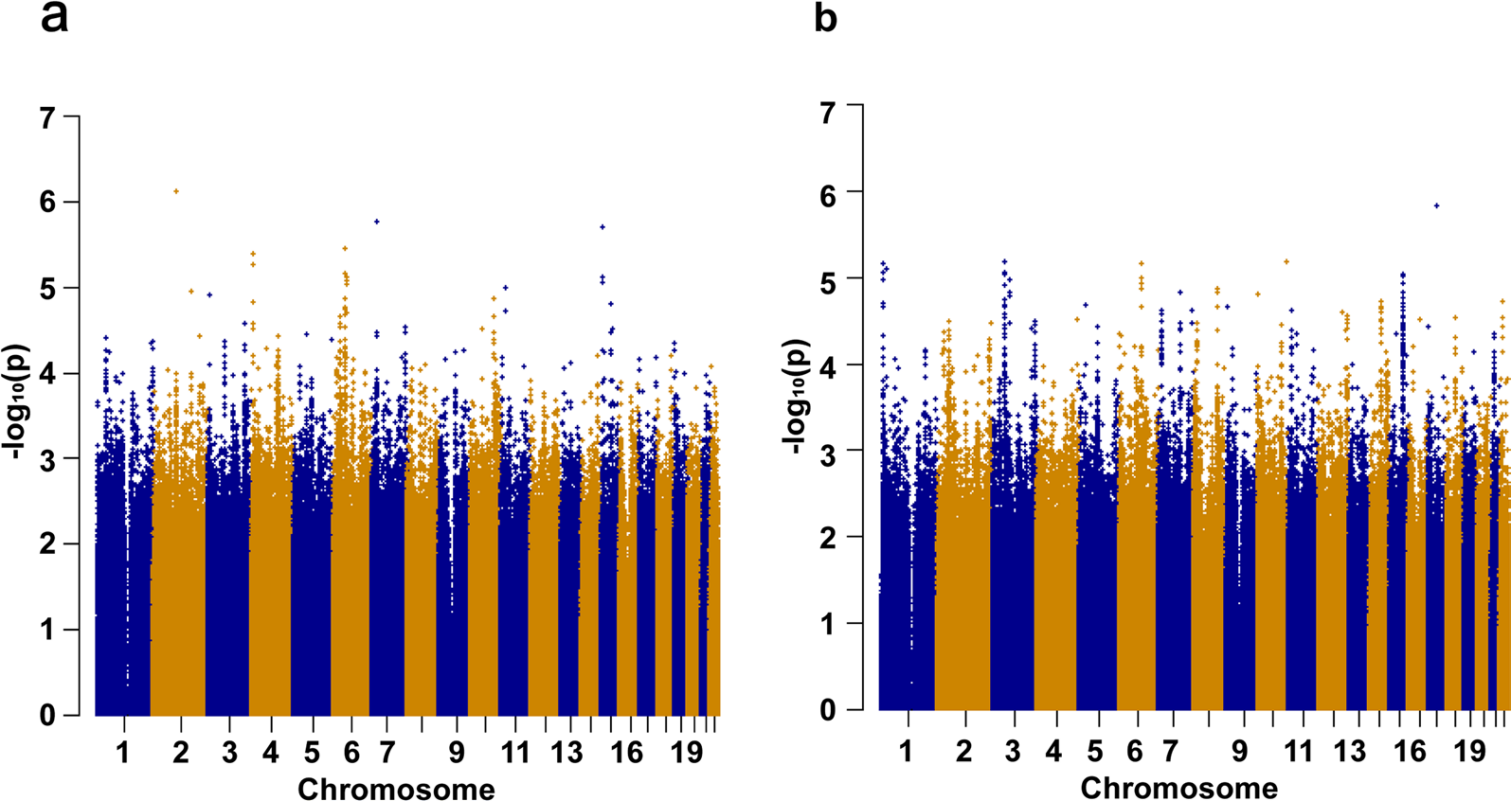
Genome-wide association study results in discovery cohort. Manhattan plot showed the genome-wide association results for primary lesion (**A**) and positive lymph node efficacy (**B**). Association P values are expressed as −log10(p). P values were from multivariate logistic regression analysis

Q-Q plots showed that the expected P-value distributions for all phenotypes were only slightly deviated in the tail region (Additional file 1: Fig. S1). The genomic inflation factor λ for the efficacy of primary lesions and positive lymph nodes after radiotherapy were 1.013 and 1.011 respectively, which were close to 1. In addition, the PCA results showed that there were no deviations from the sample for each efficacy (Additional file 1: Fig. S2). These results suggest that the patient population is ethnically homogeneous and that the observed differences in association analysis are not caused by the internal structure of the population. The Manhattan plot in (Fig. 2a, b) shows the results for both efficacy results of the discovery stage. We further selected significant variants with P < 1 × 10^− 6^ for genotyping in the validation stage samples. A total of 19 loci entered the validation stage. For the SNP locus rs6414584 screened in the discovery stage, the mass array genotyping was not performed again in the validation stage as it did not pass the conditions of quality control, and the P value and OR value in the validation stage were not given in the table. 18 loci were finally subjected to association analysis in the validation stage. For the efficacy of primary lesions, no SNPs were successfully replicated as being associated with the specific efficacy in the validation cohort (Additional file 1: Table S2). For the efficacy of positive lymph nodes, rs11130424 was successfully validated by showing statistical significance (Additional file 1: Table S3). We further confirmed the effect of rs11130424 on the sensitivity of radiotherapy for NPC with another validation cohort, and statistically significant difference still existed for rs11130424. After combination of the two validation stages, rs11130424 showed a highly statistically significant difference (Table 2). We suggest that rs11130424 may have a correlation with the efficacy of positive lymph nodes after radiotherapy. Thus, it was conducted further analysis.

**Table 2:** The final validated SNP associated with positive lymph node efficacy in nasopharyngeal carcinoma patients after radiotherapy on CACNA2D3 gene

Characteristics	rs11130424	
	*P* value	OR (95%CI)
Discovery stage	8.71×10^-6^	2.53 (1.68-3.81)
Validation stage 1	0.046	1.26 (1.00-1.59)
Validation stage 2	1.33×10^-4^	2.33 (1.51-3.62)
Combined stage	1.38×10^-5^	1.70 (1.34-2.15)

### Stratification analysis

**Fig. 3:**
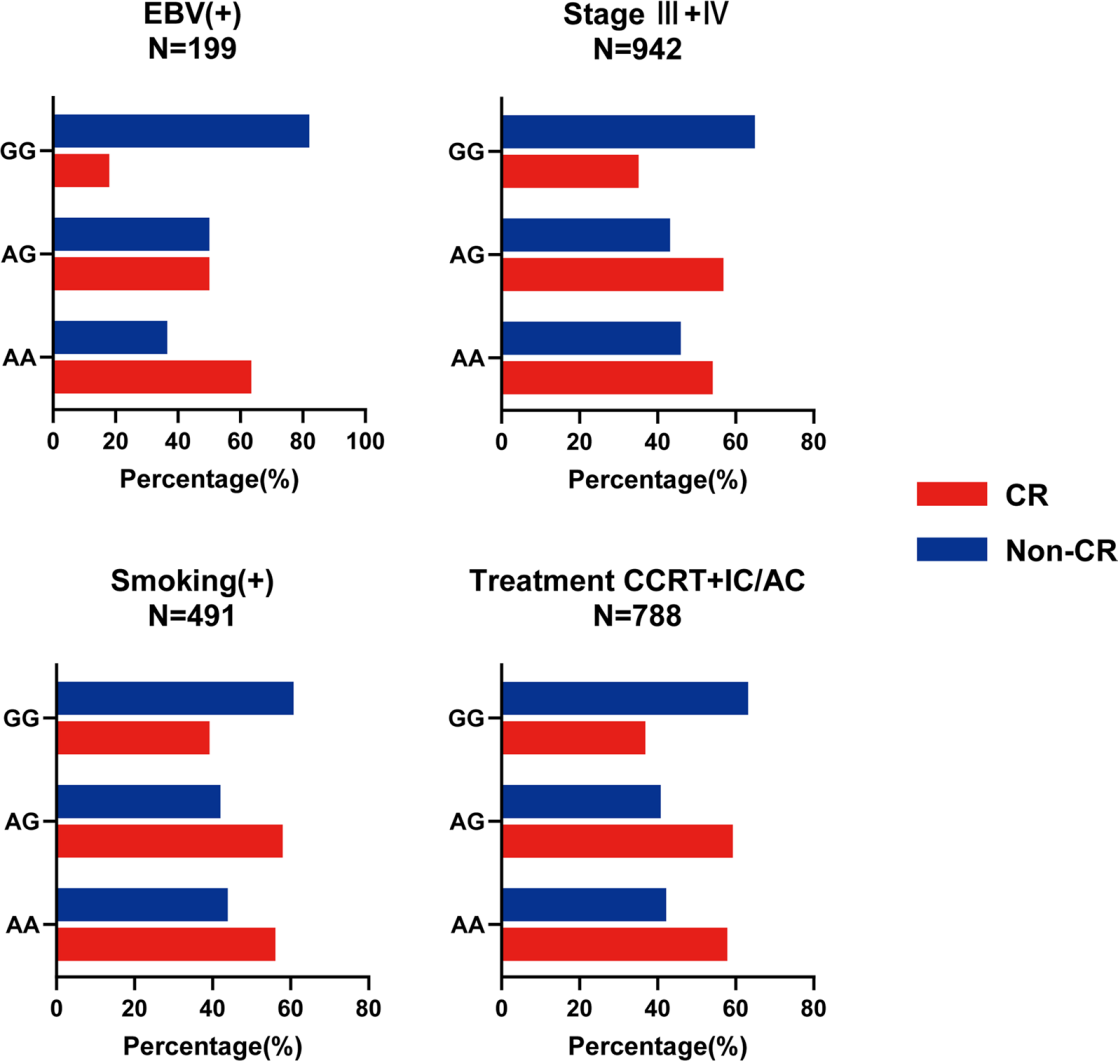
Patients distribution in different rs11130424 genotypes according to the radiotherapy response in total and stratified subgroups

As previously reported, clinical factors may also influence the sensitivity of radiotherapy. Therefore, we conducted a stratified analysis of rs11130424. Patients were grouped differently according to smoking status, EBV concentration, clinicopathological grade and treatment regimen. Correlations between the rs11130424 locus and these clinical factors were calculated. Clinical characteristics showed some significant influence on the genetic correlation of rs11130424. As shown in Table 3, for smoking status, EBV infection, clinical stage and treatment scheme, the association existed in subgroups of EBV positive, smoking, late stage (III and IV) and patients received both concurrent chemoradiotherapy and induction/adjuvant chemotherapy. This result suggests that clinical factors may influence the results of radiotherapy sensitivity analysis. For example, more than half of patients who were homozygous mutant with late stage (III and IV) were effectively managed after receiving radiotherapy, and the same trend was observed in the subgroup of patients who smoked and received both chemoradiotherapy and induction/adjuvant chemotherapy (Fig. 3).

**Table 3:** Stratified analysis of the association between the efficacy of positive lymph nodes and rs11130424

Characteristics	rs11130424	
	*P* value	OR (95% CI)
Smoking status		
Nonsmoker	0.107	1.234 (0.955–1.595)
Smoker	0.0103	1.018 (0.788–1.316)
EBV		
Positive	3.175 × 10^− 4^	2.107 (1.401–3.170)
Negative	0.987	0.998 (0.822–1.213)
Clinical stage		
I + II	0.228	1.368 (0.945–2.975)
III + IV	0.0126	1.837 (1.046–3.223)
Treatment scheme		
CCRT	0.551	1.064 (0.868–1.305)
CCRT + IC/AC	3.403 × 10^− 3^	2.214 (1.296–3.783)

Based on these results, we concluded that rs11130424 correlates with radiotherapy response in patients of EBV positive, smoking, late stage (III and IV) and receiving both concurrent chemoradiotherapy and induction/adjuvant chemotherapy.

### Gene mapping associated with radiotherapy efficacy

**Fig. 4:**
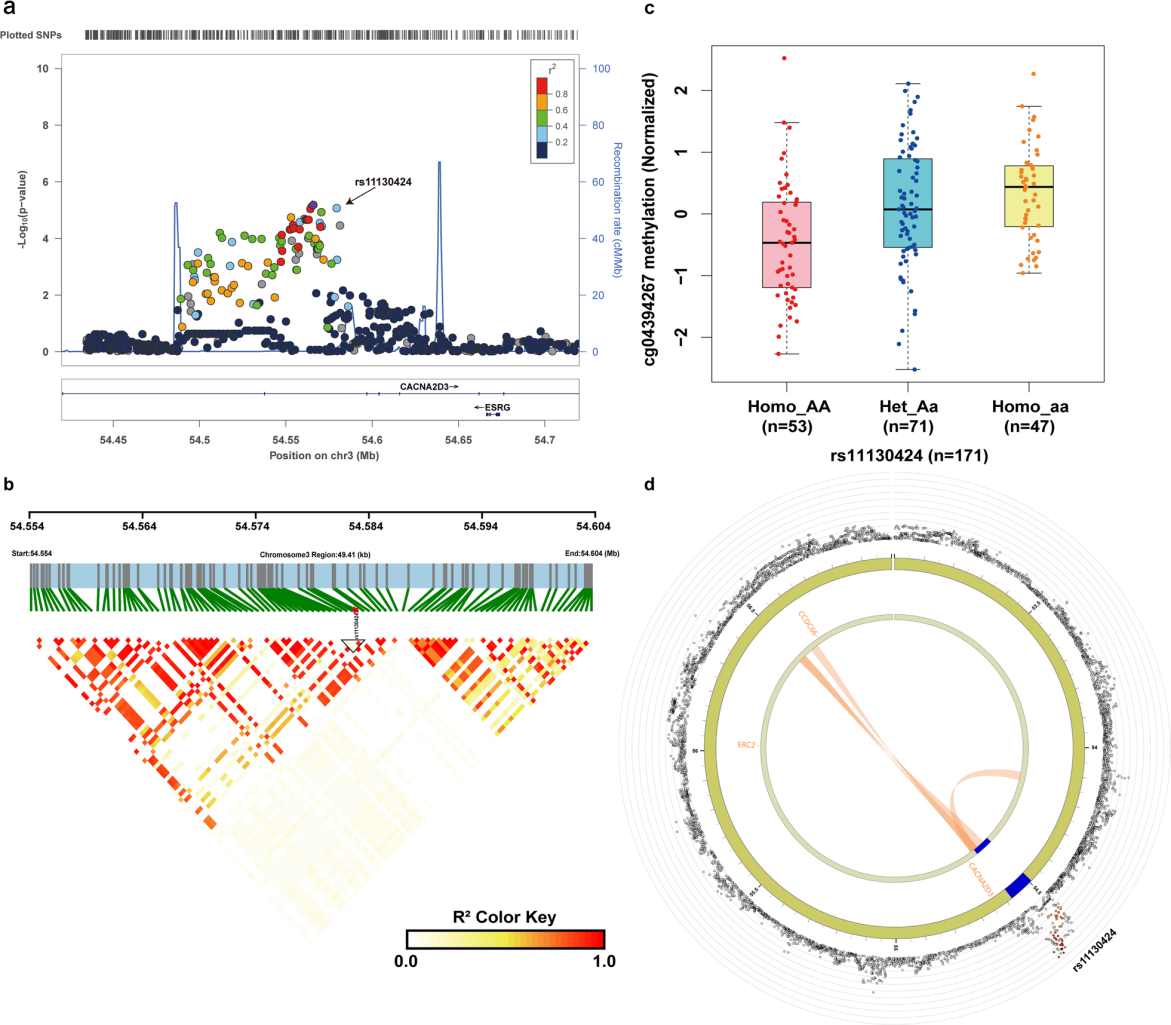
Gene mapping and annotation analysis of rs11130424. **A** The regional plot for rs11130424. –log_10_ P-values are shown along the left y-axis, and the right y-axis corresponds to recombination rate, plotted as a blue line. The x-axis indicates chromosomal position. The variants are colored according to their correlation (r^2^) with the lead variant (see legend). **B** Linkage disequilibrium (LD) analysis spanning the physical position from 54.554 to 54.604 Mb of chromosome 3. The color key indicates the level of LD (r^2^) between variants in 1000 Genomes Project East Asian populations. **C** The association between different genotypes at rs11130424 and methylation levels at the significant CpG probe cg04394267. The level of methylation at the CpG probe is shown as the β-value. The box plots show the distribution of the methylation levels in each genotype category with error bars representing the 25% and 75% quantiles. **D** 3D chromatin interaction and eQTL analysis of rs11130424. Circos plot showing genes on chromosome 3 interacted with rs11130424 by 3D chromatin interaction. The most outer layer showed the –log10(P values) of chromosome 3 in the GWAS analysis. Only SNPs with P < 0.05 are displayed. Links colored orange are chromatin interactions

To learn the functional consequence of rs11130424, we performed a fine-mapping and functional annotation. As indicated in Fig. 4a, rs11130424 were located in the chromosome 3p21.1. We first performed a linkage disequilibrium analysis using LDBlockShow. Analysis of a block spanning 49.41 kb showed that rs11130424 was not in linkage with any proximal SNPs (Fig. 4b). We further analyzed the eQTL and meQTL profiles of this locus in GTEX and Pancan-meQTL databases. Although rs11130424 was located in the intron region of calcium voltage-gated channel auxiliary subunit alpha2delta 3(CACNA2D3), eQTL analysis did not show that it had an effect with the expression of CACNA2D3 (Additional file 1: Fig. S3). Moreover, the methylation level of the locus differed significantly among genotypes in endometrial carcinoma (P = 4.87 × 10^− 6^, Fig. 4c). Whether the corresponding methylation site affects the expression of gene needs to be further explored. With the development of three-dimensional genomics, chromatin loops can spatially bring enhancers and promoters closer together, allowing enhancers to bind to promoters and play a role in regulating genes. 3D chromatin interaction indicated that they interacted with coiled-coil domain containing 66 (CCDC66) (Fig. 4d).

### Ethnic frequency analysis

Considering that it was in Chinese NPC patients that the rs11130424 locus was identified, we explored its ethnic differences by comparing MAF in five major populations. As shown in Additional file 1: Fig. S4 in the Supplement, the locus showed significant ethnic differences with a high frequency in East Asian populations, further indicating the potential and value of the rs11130424 locus as a biomarker in Chinese populations.

## Discussion

In our study, we explored the genetic variants associated with radiotherapy response using GWAS in a total of 1166 NPC patients. We identified a novel germline mutation of rs11130424 in the CACNA2D3 gene. The G allele carriers were more resistant to radiotherapy. Further stratified analysis showed that there was also a correlation existed in subgroups of EBV positive, smoking, late stage (III and IV) and patients received both concurrent chemoradiotherapy and induction/adjuvant chemotherapy. The efficacy was better in minor allele carriers of rs11130424 than major allele. Rs11130424 is located on the intron region of the CACNA2D3 gene, and the mapped genes were CACNA2D3 and CCDC66.

Radiotherapy is the main treatment for NPC, but there is some individual difference in the efficacy of treatment for patients receiving radiotherapy, the exact mechanism of which is not fully explained. For nasopharyngeal carcinoma, little is known about the genetic variants associated with radiotherapy sensitivity. Yang Z et al. showed an association between variants in autophagy-associated genes (ATG) and radiotherapy efficacy in nasopharyngeal carcinoma. ATG10 rs10514231, rs1864183 and rs4703533 were significantly associated with poorer radiotherapy efficacy at the primary site and positive lymph nodes, while ATG16L2 rs10898880 was positively associated with radiotherapy efficacy [14]. A number of genetic variants have been reported to be associated with radiotherapy response for cancer, including XRCC1, X-ray repair cross complementing 2 (XRCC2), xeroderma pigmentosum group D (XPD), and EGFR [15, 16]. However, these studies have mainly been conducted by candidate gene association strategies, focusing on DNA repair genes. GWAS provides a powerful strategy to discover novel genetic variants. Therefore, we conducted a GWAS study to identify genetic variants associated with radiotherapy sensitivity in NPC patients at a genome-wide level. After a validation stage of association analysis, we eventually found that rs11130424 on chromosome 3p21.1 was associated with response to radiotherapy. This locus is a novel finding and has not been previously reported in nasopharyngeal carcinoma or other cancers.

To date, genome-wide association analysis studies of radiotherapy in head and neck cancers, including nasopharyngeal carcinoma, have focused more on radiotherapy toxicity, and they have revealed a number of genetic variants associated with multiple toxicities, including acute xerostomia [17], radiotherapy-induced mucositis [18], and radiotherapy-induced brain injury. [19] Previously, we also performed a GWAS study of multiple acute radiotherapy toxicities in 1084 patients with nasopharyngeal carcinoma. Rs6711678, rs4848597, rs4848598 and rs2091255 on chromosome 2q14.2 and rs5845477 are novel risk loci for skin toxicity and dysphagia [20]. In this study, we provided the first GWAS study for radiotherapy response in NPC patients. We found that rs11130424 on chromosome 3p21.1 was correlated with radiotherapy sensitivity. Gene mapping and annotation indicated its implicated gene was CACNA2D3 and CCDC66.

**Fig. 5:**
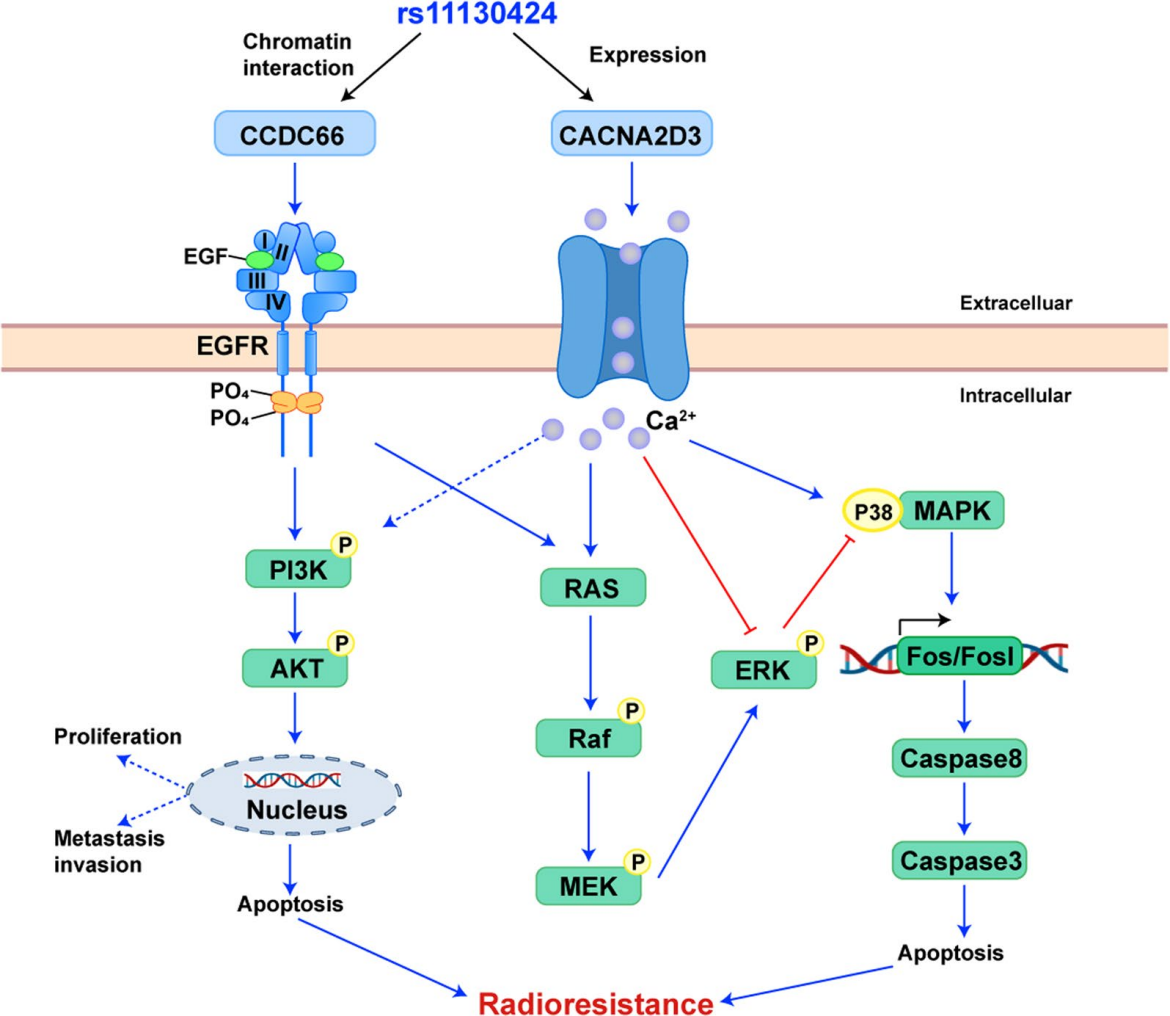
The potential molecular mechanisms of correlation between rs11130424 and efficacy after radiotherapy. Rs11130424 implicated expression of CACNA2D3, which regulates PI3K/AKT and Ras/MARK pathways. In addition, 3D chromatin interaction indicated that they interacted with CCDC66, which regulate tumor proliferation, metastasis, angiogenesis and apoptosis. *PI3K* phosphatidylinositol 3-kinase; MEK: kinse-ERK kinase, *ERK* extracellular signal-regulated kinase, *MARK* mitogen-activated protein kinase, *caspase 8* apoptosis-related cysteine peptidase 8, *caspase 3* apoptosis-related cysteine peptidase 3

CACNA2D3 encodes a subunit of the voltage-dependent calcium channel complex that regulates intracellular Ca2 + influx. It was revealed that CACNA2D3 promoted radiation-induced apoptosis by regulating intracellular calcium ion concentration; and affected the expression of several genes, correlating with the activity of multiple signaling pathways in oesophageal cancer (Fig. 5); in particular, CACNA2D3 inhibited the phosphorylation of PI3K and AKT [21]. Studies have demonstrated that activation of PI3K signaling pathways is closely associated with the development of NPC [22]. As the PI3K/AKT signaling pathway is highly activated in NPC and is closely associated with tumor radiotherapy sensitivity, CACNA2D3 is involved in regulating PI3K, and we speculate that this may be the mechanism by which variants in the CACNA2D3 gene are associated with the occurrence of radiotherapy resistance in patients. In addition, it has been reported that increased levels of Ca2 + in the cytoplasm activate the MAPK cascade [23], and Ras, Raf, MEK and ERK proteins are key factors in this pathway. Studies have shown that CACNA2D3/CA2 + induces phosphorylation of p38 MAPK in endometrial cancer, which in turn induces apoptosis in tumor cells [24]. CACNA2D3 may influence radiotherapy resistance by regulating the PI3K/AKT and Ras/MARK pathways (Fig. 5).

CCDC66 is a member of the coiled-coil structural domain (CCDC) proteins, which act primarily as oncogenes to regulate tumor proliferation, metastasis, angiogenesis and apoptosis. Although there is no evidence that CCDC66 is directly involved in resistance to radiotherapy, another protein in the family, CCDC86, promotes the proliferation and invasive migration of NPC cells by regulating the activation of the PI3K/AKT pathway by EGFR. EGFR is a key upstream regulator of the PI3K/AKT pathway, and EGFR phosphorylation can indirectly activate PI3K [25]. It has been implicated that the EGFR-PI3K/AKT pathway induces NPC cell senescence and suppresses the tumor stem cell phenotype [26, 27]. We hypothesized that CCDC66 may have a similar regulatory mechanism in nasopharyngeal carcinoma radiotherapy resistance (Fig. 5).

In addition, the gene expression profile and clinical characteristics of GSE48503 were obtained from the GEO database (https://www.ncbi.nlm.nih.gov/geo/query/acc.cgi) for download. CACNA2D3 and CCDC66 genes were also found among the differentially expressed gene profiles of radiation-resistant and radiation-sensitive NPC cells, which also reaffirmed the reliability of our results (Additional file 1: Fig. S5).

The present study has several limitations. Firstly, the sample size of our study did not have sufficient power to conclusively demonstrate the association between rs11130424 and radiotherapy efficacy, and also reduced the possibility of identifying other genetic variants. Another limitation is that the exact mechanism by which methylation at the rs11130424 locus affects the phenotype is unclear, as it is located in the intron region of CACNA2D3 and the role of methylation in the gene has not been fully established. And in vitro functional studies are needed to validate it. Finally, more newly identified genetic variants can be combined with clinical factors to establish a comprehensive mathematical model for predicting individual efficacy in NPC in the future, thus providing a reference for individualized clinical treatment.

## Supplementary Information

**Additional file 1:** **Figure S1.** Quantile–quantile (QQ) plot of observed association P-values (y-axis) against expected P-values (x-axis) in the discovery stage. X-axis represents –log10 expected P-values and Y-axis represents –log10 observed P-values. **Figure S2.** Distribution of samples according to PCA analysis in discovery stage. **Figure 3.** eQTL violin plot from GTEx displaying significant association between rs11130424 and the expression of CACNA2D3 within the whole blood. **Figure S4.** The MAF of rs11130424 in different ethnic populations. SAS: South Asian, EUR: Europe, EAS: East Asian, AMR: American, AFR: African. **Figure S5. **Volcano plot showed differentially expressed genes in the sensitive and resistant groups. **Table S1. **Characteristics of NPC patients involved in primary lesion efficacy association analysis. **Table S2** Characteristics of NPC patients involved in positive lymph node efficacy association analysis. **Table S3.** SNPs significantly associated with primary lesion efficacy in nasopharyngeal carcinoma patients after radiotherapy. **Table S4.** SNPs significantly associated with positive lymph node efficacy in nasopharyngeal carcinoma patients after radiotherapy.

## Data Availability

The datasets and code that support the findings of this study are available from the corresponding author on reasonable request.

## Abbreviations

RT: Radiotherapy
NPC: Nasopharyngeal carcinoma
GWAS: Genome-wide association analysis
XRCC1: X-ray repair cross complementing 1
EGFR: Epidermal growth factor receptor
IMRT: Intensity Modulated Radiation Therapy
GTV: Total tumor volume
CTV: Clinical target volume
PTV: Planned target volume
RECIST: Response Evaluation Criteria in Solid Tumors
CR: Complete remission
PR: Partial remission
SD: Stable disease
PD: Progressive disease
QC: Quality control
PCA: Principal component analysis
eQTL: Expression quantitative trait loci
meQTL: DNA methylation quantitative trait loci
GTEx: Genotype-Tissue Expression project
FUMA: Functional mapping and annotation
OR: Odds ratio
CI: Confidence intervals
BMI: Body Mass Index
CACNA2D3: Calcium voltage-gated channel auxiliary subunit alpha2delta 3
CCDC66: Coiled-coil domain containing 66
ATG: Autophagy-associated genes
XRCC2: X-ray repair cross complementing 2
XPD: Xeroderma pigmentosum group D
CCDC: Coiled-coil structural domain
