# Supplementary material for: A two-stage genome-wide association study identifies novel germline genetic variations in CACNA2D3 associated with radiotherapy response in nasopharyngeal carcinoma
Source: J Transl Med. 2023 Jan 9;21:11. doi: 10.1186/s12967-022-03819-4 (PMC9830790; doi:10.1186/s12967-022-03819-4)
Supplement: Supplementary file 1 — Additional file 1: Figure S1. Quantile–quantile (QQ) plot of observed association P-values (y-axis) against expected P-values (x-axis) in the discovery stage. X-axis represents –log10 expected P-values and Y-axis represents –log10 observed P-values. Figure S2. Distribution of samples according to PCA analysis in discovery stage. Figure 3. eQTL violin plot from GTEx displaying significant association between rs11130424 and the expression of CACNA2D3 within the whole blood. Figure S4. The MAF of rs11130424 in different ethnic populations. SAS: South Asian, EUR: Europe, EAS: East Asian, AMR: American, AFR: African. Figure S5. Volcano plot showed differentially expressed genes in the sensitive and resistant groups. Table S1. Characteristics of NPC patients involved in primary lesion efficacy association analysis. Table S2 Characteristics of NPC patients involved in positive lymph node efficacy association analysis. Table S3. SNPs significantly associated with primary lesion efficacy in nasopharyngeal carcinoma patients after radiotherapy. Table S4. SNPs significantly associated with positive lymph node efficacy in nasopharyngeal carcinoma patients after radiotherapy. [file 12967_2022_3819_MOESM1_ESM.docx]

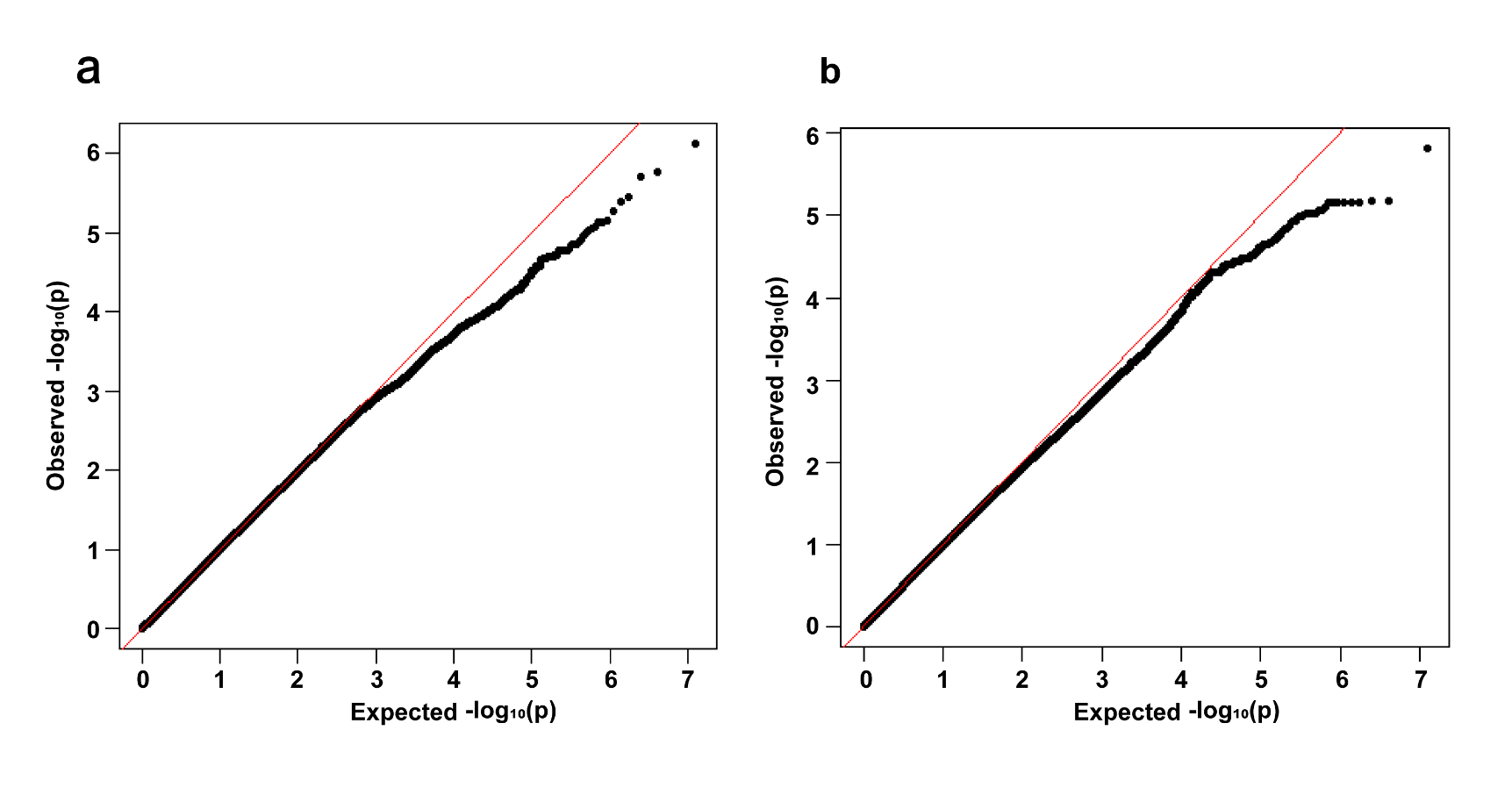
Additional file 1: **Figure S1.** Quantile–quantile (QQ) plot of observed association P-values (y-axis) against expected P-values (x-axis) in the discovery stage

X-axis represents -log10 expected P-values and Y-axis represents -log10 observed P-values.


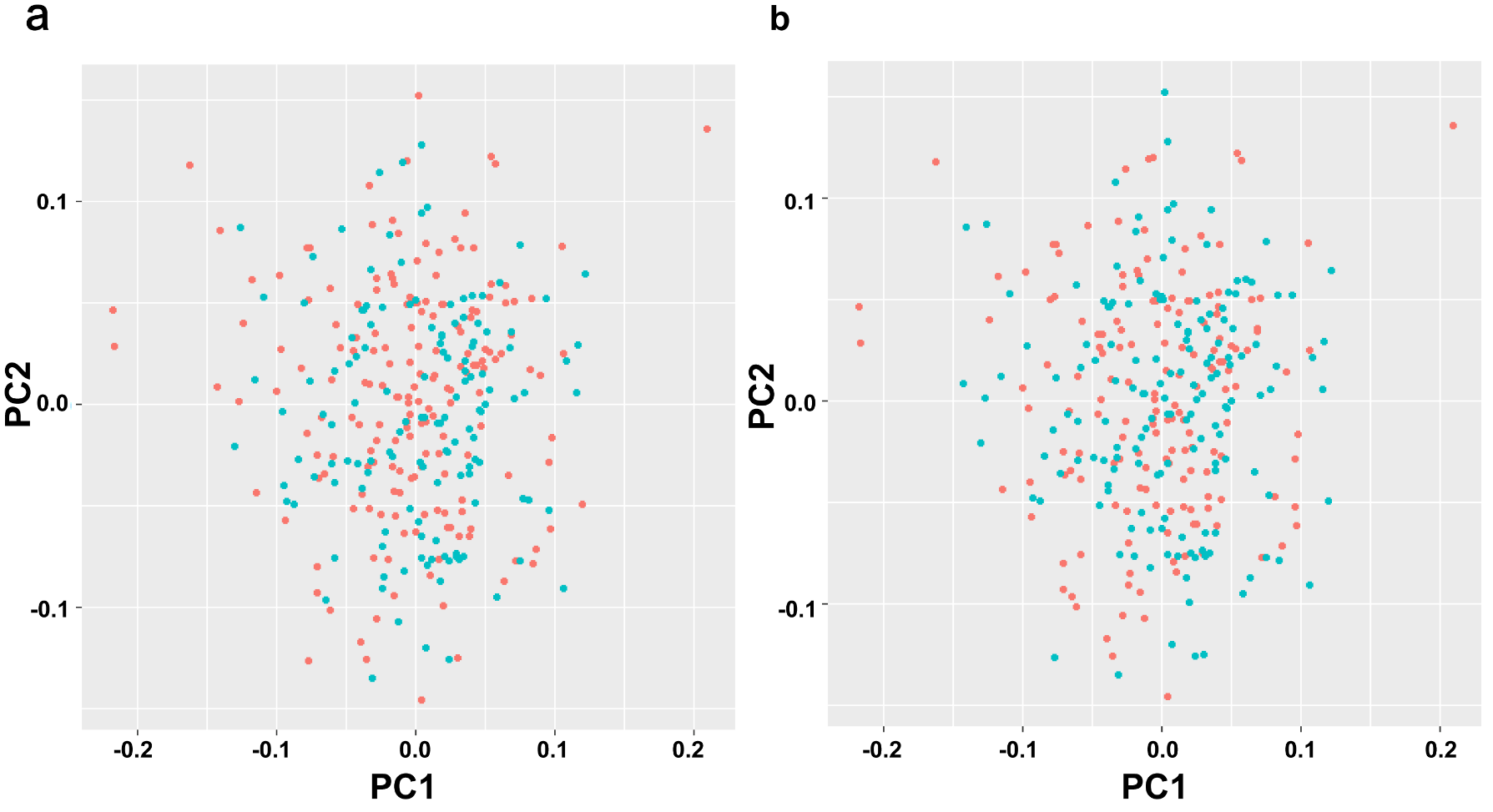
 Additional file 1: **Figure S2.** Distribution of samples according to PCA analysis in discovery stage


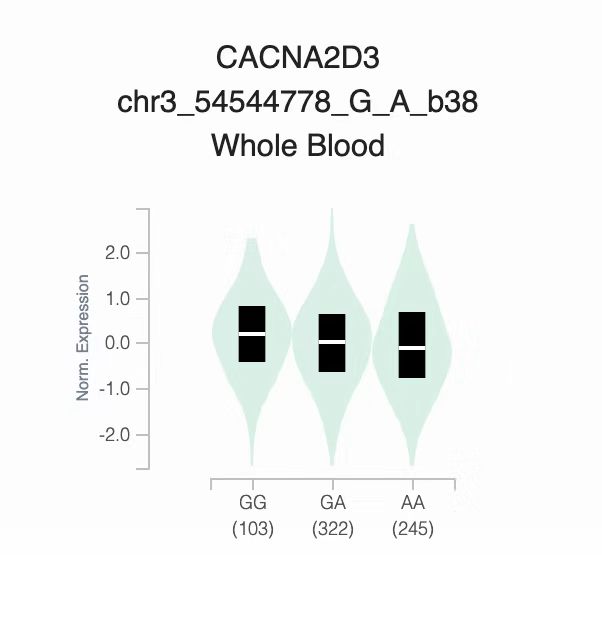
 Additional file 1: **Figure S3.** eQTL violin plot from GTEx displaying significant association between rs11130424 and the expression of CACNA2D3 within the whole blood


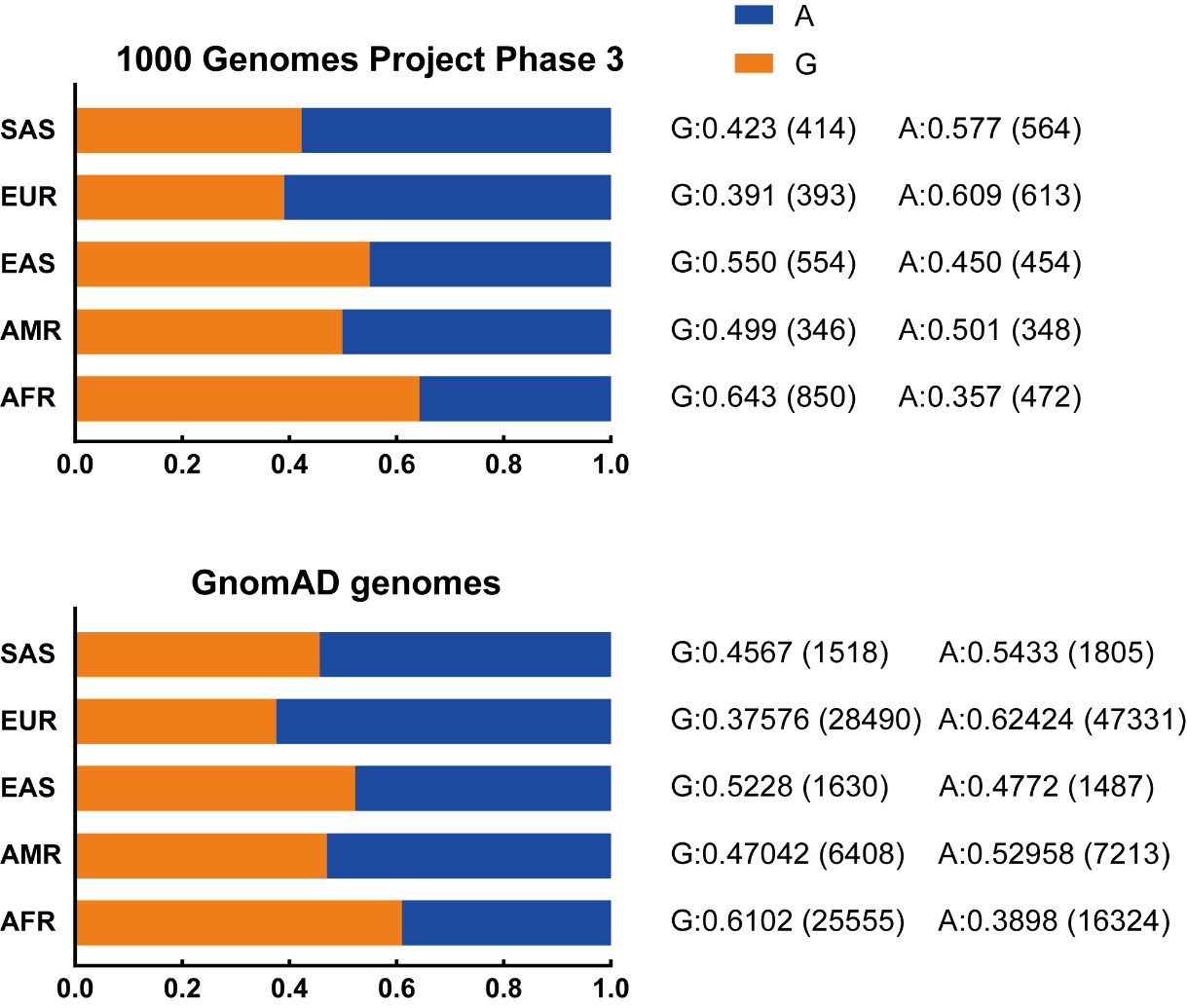
 Additional file 1: **Figure S4.** The MAF of rs11130424 in different ethnic populations. SAS: South Asian, EUR: Europe, EAS: East Asian, AMR: American, AFR: African

Additional file 1: **Figure S5.** Volcano plot showed differentially expressed genes in the sensitive and resistant groups


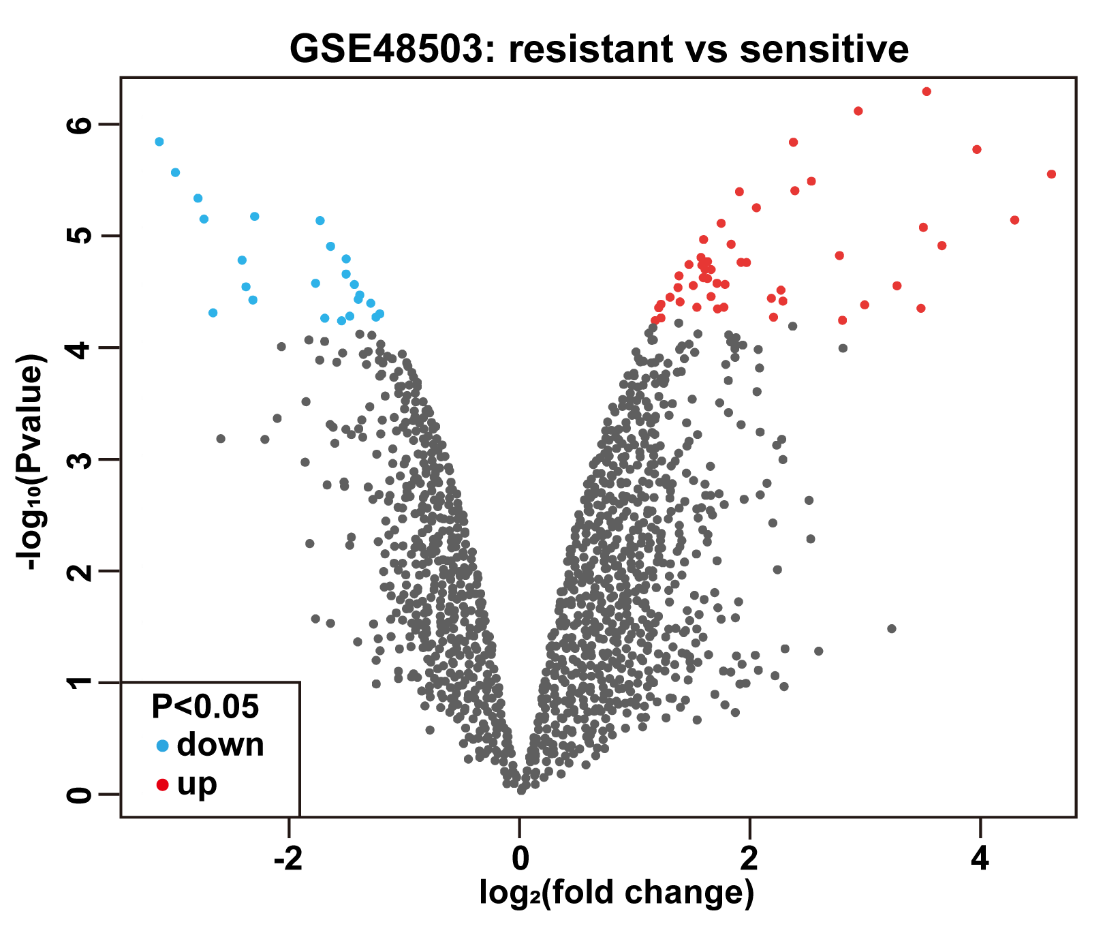


The log2(fold change) is plotted on the x-axis, and the negative log10 (P-value) is plotted on the y-axis. The represented genes have an FDR lower than 0.05.

**Additional file 1: Table S1** Characteristics of NPC patients involved in primary lesion efficacy association analysis

| **Characteristics** | **Discovery stage** | | | **Validation stage 1** | | | **Validation stage 2** | | | **Combined stage** | | |
| --- | --- | --- | --- | --- | --- | --- | --- | --- | --- | --- | --- | --- |
|  | CR | Non-CR | *P* value | CR | Non-CR | *P* value | CR | Non-CR | *P* value | CR | Non-CR | *P* value |
|  | N=182 | N=137 |  | N=146 | N=516 |  | N=30 | N=155 |  | N=358 | N=808 |  |
| **Age (Mean ± SD)** | 50.64 ± 11.14 | 50.90 ± 12.99 | 0.851 | 46.95 ± 9.37 | 47.77 ± 10.45 | 0.3966 | 50.5 ± 12.23 | 51.41 ± 12.14 | 0.9464 | 49.14 ± 10.69 | 49.22 ± 10.85 | 0.8965 |
| **Gender** |  |  |  |  |  |  |  |  |  |  |  |  |
| Male | 121 | 94 | 0.7185 | 103 | 385 | 0.3371 | 21 | 110 | 0.8333 | 245 | 589 | 0.2632 |
| Female | 61 | 43 |  | 43 | 130 |  | 9 | 45 |  | 113 | 218 |  |
| **BMI (Mean ± SD)** | 22.53 ± 3.49 | 22.25 ± 3.7 | 0.4868 | 23.22 ± 3.36 | 23.17 ± 3.24 | 0.8294 | 23.69 ± 3.20 | 23.50 ± 3.29 | 0.8059 | 22.9± 3.43 | 23.08 ± 3.32 | 0.6047 |
| **Smoking status** |  |  |  |  |  |  |  |  |  |  |  |  |
| Smoker | 87 | 59 | 0.4279 | 68 | 252 | 0.5731 | 11 | 63 | 0.7959 | 166 | 374 | 0.05331 |
| Nonsmoker | 95 | 78 |  | 78 | 256 |  | 17 | 77 |  | 190 | 411 |  |
| NR | 0 | 0 |  | 0 | 7 |  | 2 | 15 |  | 2 | 22 |  |
| **Drinking status** |  |  |  |  |  |  |  |  |  |  |  |  |
| Drinker | 48 | 23 | 0.05648 | 44 | 183 | 0.1419 | 15 | 80 | 0.6503 | 107 | 286 | 0.01552 |
| Non-drinker | 134 | 114 |  | 102 | 325 |  | 7 | 45 |  | 243 | 484 |  |
| NR | 0 | 0 |  | 0 | 7 |  | 8 | 30 |  | 8 | 37 |  |
| **EBV** |  |  |  |  |  |  |  |  |  |  |  |  |
| Positive | 81 | 53 | 0.4819 | 39 | 114 | 0.2291 | 13 | 75 | 0.03754 | 133 | 242 | 0.05434 |
| Negative | 100 | 81 |  | 107 | 395 |  | 10 | 68 |  | 217 | 544 |  |
| NR | 1 | 2 |  | 0 | 6 |  | 7 | 12 |  | 8 | 20 |  |
| **Clinical stage** |  |  |  |  |  |  |  |  |  |  |  |  |
| I | 6 | 3 | 0.559 | 5 | 13 | 0.8947 | 1 | 1 | 0.09819 | 12 | 17 | 0.2881 |
| II | 16 | 8 |  | 12 | 43 |  | 5 | 7 |  | 33 | 58 |  |
| III | 65 | 56 |  | 66 | 236 |  | 14 | 78 |  | 145 | 370 |  |
| IV | 94 | 69 |  | 55 | 203 |  | 10 | 68 |  | 159 | 340 |  |
| NR | 0 | 1 |  | 8 | 20 |  | 0 | 1 |  | 8 | 22 |  |
| **Treatment scheme** |  |  |  |  |  |  |  |  |  |  |  |  |
| RT alone | 33 | 28 | 0.7766 | 2 | 10 | 0.07593 | 3 | 17 | 0.9213 | 38 | 55 | 0.32 |
| RT+IC/AC | 3 | 1 |  | 9 | 30 |  | 2 | 17 |  | 14 | 48 |  |
| CCRT | 56 | 42 |  | 3 | 40 |  | 6 | 29 |  | 65 | 111 |  |
| CCRT+IC/AC | 90 | 65 |  | 127 | 404 |  | 19 | 93 |  | 236 | 562 |  |
| RT+other treatment* | 0 | 1 |  | 5 | 31 |  | 0 | 0 |  | 5 | 32 |  |

**Additional file 1: Table S2** Characteristics of NPC patients involved in positive lymph node efficacy association analysis

| **Characteristics** | **Discovery stage** | | | **Validation stage** | | | **Validation stage 2** | | | **Combined stage** | | |
| --- | --- | --- | --- | --- | --- | --- | --- | --- | --- | --- | --- | --- |
|  | CR | Non-CR | *P* value | CR | Non-CR | *P* value | CR | Non-CR | *P* value | CR | Non-CR | *P* value |
|  | N=165 | N=96 |  | N=227 | N=421 |  | N=73 | N=102 |  | N=465 | N=619 |  |
| **Age (Mean ± SD)** | 51.51 ± 11.60 | 48.73 ± 13.27 | 0.07791 | 48.31 ± 9.90 | 47.41 ± 9.96 | 0.2743 |  |  |  |  |  |  |
| **Gender** |  |  |  |  |  |  |  |  |  |  |  |  |
| Male | 110 | 63 | 0.8925 | 162 | 316 | 0.3493 | 50 | 74 | 0.6142 | 322 | 453 | 0.174 |
| Female | 55 | 33 |  | 65 | 105 |  | 23 | 28 |  | 143 | 166 |  |
| **BMI (Mean ± SD)** | 22.39 ± 3.64 | 22.69 ± 3.05 | 0.5 | 22.78 ± 3.12 | 23.40 ± 3.35 | 0.127 |  |  |  |  |  |  |
| **Smoking status** |  |  |  |  |  |  |  |  |  |  |  |  |
| Smoker | 75 | 41 | 0.6996 | 109 | 206 | 0.9557 | 30 | 44 | 0.05786 | 214 | 291 | 0.1756 |
| Nonsmoker | 90 | 55 |  | 116 | 212 |  | 39 | 56 |  | 245 | 323 |  |
| NR | 0 | 0 |  | 2 | 3 |  | 8 | 2 |  | 10 | 5 |  |
| **Drinking status** |  |  |  |  |  |  |  |  |  |  |  |  |
| Drinker | 40 | 16 | 0.1626 | 70 | 154 | 0.3462 | 16 | 18 | 0.7695 | 126 | 188 | 0.4199 |
| Non-drinker | 125 | 80 |  | 155 | 264 |  | 19 | 30 |  | 299 | 374 |  |
| NR | 0 | 0 |  | 2 | 3 |  | 38 | 54 |  | 40 | 57 |  |
| **EBV** |  |  |  |  |  |  |  |  |  |  |  |  |
| Positive | 70 | 53 | 0.0755 | 49 | 101 | 0.7761 | 30 | 57 | 0.06606 | 149 | 211 | 0.4111 |
| Negative | 92 | 43 |  | 177 | 318 |  | 43 | 45 |  | 312 | 406 |  |
| NR | 3 | 0 |  | 1 | 2 |  | 0 | 0 |  | 4 | 2 |  |
| **Clinical stage** |  |  |  |  |  |  |  |  |  |  |  |  |
| I | 2 | 0 | 0.1198 | 6 | 11 | 0.1689 | 0 | 0 | 0.1075 | 8 | 11 | 0.5818 |
| II | 13 | 4 |  | 22 | 32 |  | 6 | 5 |  | 41 | 41 |  |
| III | 70 | 32 |  | 92 | 209 |  | 42 | 46 |  | 204 | 287 |  |
| IV | 79 | 60 |  | 96 | 158 |  | 25 | 51 |  | 200 | 269 |  |
| NR | 1 | 0 |  | 11 | 11 |  | 0 | 0 |  | 12 | 11 |  |
| **Treatment scheme** |  |  |  |  |  |  |  |  |  |  |  |  |
| RT alone | 28 | 14 | 0.5708 | 5 | 6 | 0.6473 | 7 | 8 | 0.5684 | 40 | 28 | 0.00126 |
| RT+IC/AC | 2 | 2 |  | 14 | 25 |  | 9 | 12 |  | 25 | 39 |  |
| CCRT | 44 | 31 |  | 10 | 12 |  | 11 | 9 |  | 65 | 52 |  |
| CCRT+IC/AC | 91 | 48 |  | 185 | 360 |  | 46 | 73 |  | 322 | 481 |  |
| RT+other treatment* | 0 | 1 |  | 13 | 18 |  | 0 | 0 |  | 13 | 19 |  |

**Additional file 1: Table S3** SNPs significantly associated with primary lesion efficacy in nasopharyngeal carcinoma patients after radiotherapy

| CHR | SNP | Gene | Allele | MAF | Discovery stage | | Validation stage | |
| --- | --- | --- | --- | --- | --- | --- | --- | --- |
|  |  |  |  |  | *P* | OR (95%CI) | *P* | OR (95%CI) |
| 4 | rs10938685 | ABLIM2 | T>C | 0.460 | 4.05×10^-6^ | 2.44(1.67-3.57) | 0.824 | 0.97(0.74-1.27) |
| 4 | rs13136604 | ABLIM2 | T>C | 0.458 | 5.32×10^-6^ | 2.42(1.65-3.53) | 0.941 | 0.99(0.76-1.28) |
| 6 | rs5876334 | NA | T/TGT | NA | 3.51×10^-6^ | 2.90(1.85-4.55) | 0.248 | 2.33(0.53-10.19) |
| 6 | rs34759759 | NA | dupT | 0.400 | 6.94×10^-6^ | 2.78(1.78-4.34) | 0.252 | 1.20(0.88-1.64) |
| 7 | rs17317920 | ENSG00000285960 | A>G | 0.481 | 1.73×10^-6^ | 4.50(2.43-8.32) | 0.591 | 0.91(0.64-1.29) |
| 15 | rs10519577 | GABRG3 | A>G | 0.165 | 2.00×10^-6^ | 3.90(2.22-6.81) | 0.539 | 1.13(0.76-1.67) |

**Additional file 1: Table S4** SNPs significantly associated with positive lymph node efficacy in nasopharyngeal carcinoma patients after radiotherapy

| CHR | SNP | Gene | Allele | MAF | Discovery stage | | Validation stage | |
| --- | --- | --- | --- | --- | --- | --- | --- | --- |
|  |  |  |  |  | *P* | OR (95%CI) | *P* | OR (95%CI) |
| 1 | rs2131630 | TNFRSF9 | G>A | 0.394 | 6.92×10^-6^ | 2.54(1.69-3.80) | 0.209 | 1.16(0.92-1.47) |
| 1 | rs2472862 | TNFRSF9 | G>A | 0.394 | 6.92×10^-6^ | 2.54(1.69-3.80) | 0.248 | 1.15(0.91-1.46) |
| 1 | rs863171 | TNFRSF9 | C>T | 0.407 | 6.92×10^-6^ | 2.54(1.69-3.80) | 0.400 | 1.11(0.87-1.40) |
| 1 | rs12117402 | HSPG2 | T>A | 0.285 | 8.05×10^-6^ | 2.45(1.65-3.63) | 0.379 | 1.11(0.88-1.41) |
| 3 | rs6445687 | CACNA2D3 | A>C | 0.321 | 9.33×10^-6^ | 0.39(0.26-0.59) | 0.0584 | 1.26(0.99-1.59) |
| 6 | rs746964 | NA | C>T | 0.246 | 6.94×10^-6^ | 0.37(0.24-0.57) | 0.665 | 0.95(0.74-1.21) |
| 15 | rs8038666 | LINGO1 | T>C | 0.481 | 9.32×10^-6^ | 2.36(1.61-3.44) | 0.359 | 0.90(0.71-1.13) |
| 15 | rs28537849 | LINGO1 | C>G | 0.481 | 9.32×10^-6^ | 2.36(1.61-3.44) | 0.536 | 0.93(0.73-1.18) |
| 15 | rs11072669 | LINGO1 | T>A | 0.481 | 9.32×10^-6^ | 2.36(1.61-3.44) | 0.387 | 0.90(0.71-1.14) |
| 15 | rs8028520 | LINGO1 | A>C | 0.481 | 9.32×10^-6^ | 2.36(1.61-3.44) | 0.360 | 0.90(0.71-1.13) |
| 15 | rs8035384 | LINGO1 | T>G | 0.480 | 9.77×10^-6^ | 2.35(1.61-3.43) | 0.428 | 0.91(0.72-1.15) |
